# Precision Medicine and Big Data: The Application of an Ethics Framework for Big Data in Health and Research

**DOI:** 10.1007/s41649-019-00094-2

**Published:** 2019-09-30

**Authors:** G. Owen Schaefer, E Shyong Tai, Shirley Sun

**Affiliations:** 1 Centre for Biomedical Ethics, Yong Loo Lin School of Medicine, National University of Singapore, Singapore; 2 Saw Swee Hock School of Public Health, National University of Singapore, Singapore; 3 Division of Endocrinology, National University Hospital, Singapore; 4 Sociology, School of Social Sciences and Lee Kong Chian School of Medicine, Nanyang Technological University, Singapore

**Keywords:** Precision medicine, Big data, Bioethics, Genomics, Genetic discrimination

## Abstract

As opposed to a ‘one size fits all’ approach, precision medicine uses relevant biological (including genetic), medical, behavioural and environmental information about a person to further personalize their healthcare. This could mean better prediction of someone’s disease risk and more effective diagnosis and treatment if they have a condition. Big data allows for far more precision and tailoring than was ever before possible by linking together diverse datasets to reveal hitherto-unknown correlations and causal pathways. But it also raises ethical issues relating to the balancing of interests, viability of anonymization, familial and group implications, as well as genetic discrimination. This article analyses these issues in light of the values of public benefit, justice, harm minimization, transparency, engagement and reflexivity and applies the deliberative balancing approach found in the *Ethical Framework for Big Data in Health and Research* (Xafis et al. 2019) to a case study on clinical genomic data sharing. Please refer to that article for an explanation of how this framework is to be used, including a full explanation of the key values involved and the balancing approach used in the case study at the end. Our discussion is meant to be of use to those involved in the practice as well as governance and oversight of precision medicine to address ethical concerns that arise in a coherent and systematic manner.

## Background

Precision medicine is, in its broad contours, not a new approach. Clinicians have generally sought to deliver therapies and preventative care that will best suit the particular patient in front of them, taking into account all relevant contextual factors. However, big data allows for far more precision and tailoring than was ever before possible by linking together diverse datasets to reveal hitherto-unknown correlations and causal pathways. This new information can, in turn, guide the development of treatments or treatment strategies truly individualized to the person receiving them. Relevant information includes genetic data that could, for example, be used to develop new medications, predict the risk of a side effect of a drug or to establish the diagnosis of a rare disease (see Box 1).

**Table:** 

Box 1: Big data applications in precision medicine		
Basic research	Clinical research	Clinical practice
-Facilitating the discovery of molecular targets for new therapies -Facilitating the discovery of biomarkers that can be used to identify people who are likely to respond to targeted interventions or experience adverse events	-Facilitating the clinical testing of targeted therapies, novel diagnostic techniques or predictive tests	-Helping to diagnose people and target therapies at their particular molecular/behavioural profile -Establishing more effective preventative care through more accurate prediction of likely disease onset

Behavioural and lifestyle factors (such as diet, daily activities, even social media use) interact with genetic factors to cause disease; this means that data that relates to lifestyle also contains information about key determinants of risk for many of the common chronic diseases that afflict modern societies. These behavioural factors can also interact with biological factors to cause disease. Access to data related to these behavioural factors not only allows a better understanding of the biological effects but also allows us to identify behavioural changes that may mitigate the effects of biological variants on disease susceptibility.

The use of genomic data is also changing the face of drug and other therapeutic development. In the past, drugs have been developed based on knowledge of biological pathways involved in disease, often derived from experiments performed in cells or animals. Very often, these experiments fail to replicate in humans. Furthermore, drug development is limited to those biological processes that we know (or believe we know) about. The ability to survey the entire genome enables scientists to identify novel pathways involved in the disease and develop therapies that are more likely to be relevant to humans. But because these variants are often rare, or have small effect sizes, large datasets are required to make valid inferences about the role of these variants in disease. As such, it is critical to pool large datasets.

Precision medicine’s reliance on big data therefore greatly expands the scope of biomedical research, potentially to the societal level. This means engagement will similarly be required at the societal level. Attention to ethical issues by stakeholders such as researchers, data custodians, hospitals, research institutions, regulators and governance bodies, then, is not only intrinsically important (for prevention of harms, injustice and disrespect), but pragmatically essential if precision medicine is to receive the social support it needs to be successful. This is necessary at the initial point at which data is generated or accessed for precision medicine purposes, as well as over time as standing databases are maintained and unforeseen uses of genetic and other personal information takes on unexpected uses.

Here, we survey several (non-exhaustive) key topics worth consideration in the precision medicine context and the values related to them.

## Key Issues

### Balancing of Interests/Sharing of Benefits

Like any novel therapeutic approach, precision medicine should only enjoy wide uptake if it can be shown to have benefits that sufficiently outweigh the costs to develop and deploy it, as well as the risks involved. Some of this involves cost-benefit analyses that are outside the scope of this framework. But it also means that there must be careful attention given to who benefits, realistic expectations of the magnitude of benefits (avoiding hype) and how the burdens of its use will be shared and distributed.

Big data, by its nature, cuts across large sections of society. There are still crucial demographic gaps in current health and genomic datasets, though, and failure to fill those gaps is doubly problematic: First, excluded groups might be less able to take advantage of the fruits of precision medicine (as there will be less of a robust evidence base to suitably tailor treatment to them), though they will bear other indirect costs such as via taxes to develop the field. Second, included groups will have to bear some of the costs of the relatively ineffective traditional treatment approaches that excluded groups will continue to receive via public healthcare systems. So there is a need to ensure that all groups are included in the big data being used to develop precision medicine.

However, even if the precision medicine initiatives could develop cost-effective treatments with fair access across populations, a counterfactual must be considered: what research or other social priority is being de-funded to make room for precision medicine? (Bayer and Galea 2015). In other words, what are the opportunity costs? It may be that shifting resources to precision medicine displaces funding that is of more importance to neglected groups. This reflects a broader international issue of the much-discussed ‘10/90 gap’: how (at least in the 1990s) less than 10% of international research funding was in areas that account for 90% of the global disease burden (Ramsay 2001). It is impossible to assess such priority-setting dilemmas in the abstract, but one mitigation strategy would involve promoting inclusion (as described in the preceding paragraph) while ensuring that the health priorities of all populations are being met by the precision medicine initiatives that rely on their data.

There is also a need to ensure broad access to the fruits of precision medicine. Due to the variable prevalence of rare but impactful genetic variants across different populations, progress in precision medicine requires linking together databases from geographically diverse populations. But even if there are findings derived from big data relevant to a particular subgroup, it may be that the costs or infrastructure required to deploy precision medicine interventions are prohibitive for them. Already, some are raising concerns about the high costs of targeted therapies (Ciardiello et al. 2014; Goddard et al. 2012; Ferkol and Quinton 2015); even if interventions come out favourably on a cost-benefit calculation, the high absolute costs of intervention may still exclude many, especially in countries that lack universal healthcare or have tight healthcare budgets (Rehman et al. 2016). Ensuring adequate sharing of the benefits of precision medicine itself will be costly, however, and a push for generic or biosimilar therapeutics may be seen as overly aggressive if it disincentivizes high-income countries from investing in precision medicine. Therefore, careful deliberation and robust justification for reasonable and appropriate distribution of precision medicine’s benefits will be needed among researchers, institutions, government officials and the public at large (see Box 2).

**Table:** 

Box 2: Example—PCSK9 inhibitors	
In 2006, two genetic variants in a gene known as PCSK9 were identified in individuals with low levels of low density lipoprotein cholesterol (LDL-C), a key risk factor for coronary heart disease. These were low frequency variants which were specific to certain ethnicities. In this study, a variant present in 2.6% of black subjects was associated with 28% lower LDL-C and 88% lower risk of heart disease. Another variant was present in 3.2% of Caucasian subjects and was associated with 15% lower LDL-C and 47% lower risk of heart disease. Importantly, the carriers of these variants were healthy in other aspects of their lives (Cohen et al. 2006). This was taken as a priori evidence that a drug that inhibits PCSK9 would lower LDL-C and reduce heart disease in a manner that was safe over a long term. Today, PCSK9 inhibitors have proven to be effective at reducing cardiovascular disease in certain patients, particularly those who are unresponsive or intolerant to statin therapy (Chaudhary et al. 2017). Uptake of PCKS9 inhibitors, however, has been marred by its high cost—currently between USD 5000 and USD 14,000 per year of treatment, which may last for the rest of the patient’s life. This has been shown to be not cost-effective (Korman et al. 2018), and many insurers decline to cover it (Hess et al. 2017). Access, then, will depend in large part on the ability of patients to afford the high out-of-pocket costs of the treatment. This raises questions concerning the fair allocation of benefits and burdens for high-cost, beneficial targeted interventions like PCSK9. A large number of individuals contributed to PCSK9 inhibitors’ development, via use of their genetic and clinical data. This involved some risk exposure. New data is generated, and existing data is linked together and shared in the context of a big data ecosystem; while that facilitates important research, it also increases the possibility of a data breach by leading to more data in the hands of more institutions and individuals. Moreover, public willingness to contribute such data is contingent on the expectation of general social benefit. Yet in this case, the benefits only accrued to a much smaller subset who are able to afford the therapy or have access to insurance that happens to cover it (Reddy 2017).	

### Anonymization

The implications of the difficulty of anonymization in the big data era are discussed in more detail in Xafis et al. (2019). Here, we will just note that anonymization is especially difficult to guarantee in precision medicine when genomic analysis is conducted. Not only is each genome is unique to any individual but also a recent study has shown that it is possible to re-identify most European-Americans from a de-identified genomic profile by linking it up with publicly available genetic ancestry databases (Erlich et al. 2018). Going forward, precision medicine will have to adapt to a context where anonymity of genetic data and tissue cannot be guaranteed.

### Familial/Group Implications

While it is not possible to cleanly map concepts of race and ethnicity onto genetic or biological reality, there are also clearly loose correlations among race, ethnicity and genetics (Mersha and Abebe 2015). This raises the possibility that big data analytics may generate findings of statistical significance which, when reported publicly, become exaggerated or misinterpreted and reinforce certain racial or ethnic stereotypes (insofar as some early precision medicine insights will be implemented at the group, rather than individual, level). Such challenges may only emerge years or even decades after data is initially gathered, as databases are mined by more and more researchers with more and more diverse and variable questions they seek to address.

One strategy for addressing these potential group harms is for data users to engage with communities (via community leaders or organizations) to ensure that use of data and dissemination of resultant information is done in a respectful manner that minimizes risks to the community and pursue co-governance in the formation of oversight mechanisms. However, this approach must be taken with caution, as it is not always clear who does or should speak for a ‘community’ (see Xafis et al. 2019 for further discussion of group harms and co-governance).

Commercial companies may also have incentives to use big data evidence in order to market pharmaceutical products along racial or ethnic group lines. Large-scale datasets on diverse population groups can be strategically appropriated by interested parties to the detriment of the health care of individuals designated as members of said groups. For example, Kahn argues that ‘the primary forces driving the re-invention of BiDil [a drug for treating heart failure] as an ethnic drug…were legal and commercial, rather than biomedical’ (Kahn 2004, 4). Indeed, rather than receiving more affordable treatments, the targeted population found that costs increased (Sankar and Kahn 2005).

To begin with, it is not necessarily in the interests of pharmaceutical companies to develop drugs that are only effective for patients with specific biomarkers, particularly when these markers may be only present in a small subset of the whole patient population. However, pharmaceutical companies may turn to racial or ethnic targeting (and, therefore, to produce racialized medicine) in the aftermath of poor or irregular drug performance in the general population large-scale clinical trials (Sun 2017).

Even in the ‘new’ biomarker-based drug development paradigm, it remains true that pharmaceutical companies have to channel their research and development efforts to areas where positive outcomes are likely. Moreover, one of the ways in which companies try to predict success is to rely on data that reveals the frequency of a particular mutation ‘enriched’ in certain racial and ethnic populations.

The decision to use such data to commercialize along racial or ethnic lines is not ethically neutral. The impact of the reification of categories via such racialization on already marginalized groups, beyond the narrow confines of medical treatment, must be considered. When a mechanism (whether genetic, environmental or otherwise) is known, relying directly on the mechanism rather than a crude proxy of race or ethnicity (which are social constructs that relate to many features that have nothing to do with the disease or the treatment) could be more defensible.

### Genetic Discrimination

The medical relevance of big data in precision medicine, including genomics, is a double-edged sword. On the one hand, the goal of precision medicine is to harness that data to benefit patients by better tailoring treatments and preventative care to the particular patient’s characteristics. On the other hand, predictive genetic information may be used by third parties such as insurance companies in setting premiums, or employers in making hiring decisions. If big data analytics shows that certain genetic markers increase predisposition to a disease that is costly to treat, insurers (including for health, life or disability) may seek to charge higher premiums for patients with those markers or even deny coverage altogether (Tiller et al. 2019).

Charging some patients more than others based on risk of developing disease is, of course, already prevalent in the insurance market. It is standard, for instance, to adjust premiums based on age, smoking habits and family history; such strategies are actuarially just (or, at least, there is no reason why they cannot be). It may be argued that using genetic information in premium setting is no different, as a method of tailoring policyholders’ premiums to their greater or lesser risk of disease or death. And individuals using their knowledge of their genetic risk of disease to purchase more or less comprehensive insurance without disclosing that risk to insurers may in the long run force insurers to increase premiums across the board (to offset higher payouts from high-risk individuals and lower pay-ins from low-risk individuals), unless they are able to stratify premiums based on those risks (Taylor et al. 2010).

However, a number of countries such as the USA, Canada and the UK have enacted measures to limit or prevent differential treatment in insurance or employment based on genetic profiles (Krajewska 2017). In addition to the normative rationale for such protections related to justice and public benefit (see below), anti-discrimination frameworks may be defended on pragmatic grounds as well. Worries about genetic discrimination are a great source of public concern over potential harms that may arise from the use of such data and may foster reluctance to support or participate in precision medicine initiatives (Kaufman et al. 2009; Green et al. 2015; Blasimme et al. 2019). By prohibiting or mitigating such differential treatment, countries may be able to not only prevent those harms but also assuage public anxieties and help ensure that there is adequate public support for gathering and using big data in precision medicine. Other mechanisms to be considered relate to data sharing restrictions, already present in many jurisdictions, designed to prevent third parties like insurers from gaining access without consent to the raw data that could be used as a basis for differential treatment.

## Key Values

Here, we highlight six values of particular relevance to precision medicine and big data. These values are derived from the longer list found in Xafis et al. (2019). They are not meant to be exhaustive of all relevant values in this space, but are particularly relevant and useful in addressing the issues discussed above.

### Substantive

#### Harm Minimization

Potential harms from precision medicine initiatives include genetic discrimination and group reputational effects—though we should also consider that prohibiting discrimination could harm other groups by causing an increase in their insurance premiums. Harm minimization efforts may be statutory, such as with genetic anti-discrimination legislation, or structural, as with data access limitations and encryption systems.

#### Justice

A wide array of individuals have contributed to the development of precision medicine, either through their data being used or their taxes being used to fund it. Contributors’ lack of access to precision medicine innovations due to high cost or other factors, as well as lack of inclusion and inequitable prioritization, are potential injustices. The potential harms of discrimination and group stigmatization could also be considered injustices, insofar as they exacerbate existing social and power disparities.

#### Public Benefit

Precision medicine has the potential to substantially improve patient care by generating targeted therapies, preventative screening tools and more individually tailored pharmaceutical prescriptions to avoid adverse reactions. Big data can facilitate these benefits by accelerating the ability of researchers to draw the necessary insights, but that must be balanced against competing values discussed here.

### Procedural

#### Transparency

Absolute anonymity cannot be guaranteed with precision medicine, and therefore trust must be based on alternative systems of governance and protection. But that trust can only be earned if systems are sufficiently transparent, in terms of how they operate, manage and distribute individuals’ data.

#### Engagement

Both ethically and pragmatically, engagement with the public is essential for precision medicine initiatives. Ethically, it provides legitimacy to the endeavour by helping ensure other procedural values like trustworthiness (which crucially relates to public attitudes and perceptions) are met and potential pitfalls are identified. Pragmatically, it can help further support for the responsible development of precision medicine by providing a guide for what uses would be socially acceptable.

#### Reflexivity

As an innovative and potentially disruptive approach to healthcare, precision medicine initiatives must be cognizant not only of their ethical implications but also that those implications can and will shift over time. A reflexive approach to precision medicine recognizes that the actual ethical issues faced will shift depending on the context and situation on the ground. This means avoiding one-off approaches to precision medicine ethical concerns and instead consistently revisiting decisions to adapt to an ever-changing landscape.

## Case Study: Issues in Secondary Research Use of Routine Genomic Testing Data

To illustrate how some of those values can inform deliberation over ethical issues in precision medicine and big data, let us consider a case of genomic data sharing. This is a fictional case that, while at present not a reality in any major healthcare systems, is a very real possibility in the near future. NHS England, in particular, has been rapidly expanding routine genomic testing; it has already committed to offering whole genome sequencing to all children with cancer and continues the expansion of routine sequencing in other areas (O’Regan 2019).

The case is as follows: A large hospital is considering the deployment of routine whole genome sequencing in its clinics as part of continued service improvement. The primary purpose is to implement precision medicine innovations such as pharmacogenetics, targeted therapies and targeted preventative medicine into the hospital’s standard clinical care. Whole genomic sequencing is being conducted to facilitate additional discoveries and also allow for future insights to be implemented for these patients without the need for re-testing. The aim is both to reduce costs and to improve quality of care, which the hospital administration believes is a win-win for providers and patients.

Given that precision medicine is still in its early stages, the hospital would also like sequenced data to be collated into a database that can be used for secondary data research, particularly by collaborators at other institutions, in other countries around the world. This would raise the hospital’s reputation as a cutting-edge centre of research, as well as support the development of innovations that the hospital itself relies upon.

Before proceeding, the administration wishes to consider the ethical ramifications of making genomic data available for secondary research use and what reasonable steps may be taken to mitigate any potential pitfalls. Here, we will work through the balancing approach presented in Xafis et al. (2019) to better understand these issues and actions that may be taken.

### Stating the Problems and Issues

While the wisdom and practicality of routine whole genome sequencing in the clinic may be debated, such clinical management issues are somewhat outside the scope of this big data framework. We will instead focus on the further question of making the clinical data available to the big data ecosystem where rich data is shared, linked and analysed by many disparate researchers. A wide range of ethical issues arise in this context, but we will further focus on some special concerns related to the use of genomic data. For discussion of broader implications of depositing biomedical data in repositories, see the case study at the end of Xafis and Labude (2019); for discussion of issues in sharing real-world data with for-profit entities, see the case studies at the end of Lipworth (2019) and (Ballantyne and Stewart 2019).

Any sharing of genomic data involves risks to the individuals concerned (in this case, hospital patients) from a data breach. Breaches not only could be caused by malicious actors but also as a result of inadvertent disclosure on the part of holders of genetic information. For example, DNA testing service Vitagene was recently found to have inadvertently disclosed thousands of records publicly online, including genetic test results (Grant 2019). This risk may increase as data is shared with more researchers, as there are more potential sources for a breach to occur. Genomic data breaches would be concerning, in particular, because of the risk of resultant genetic discrimination if the data were to be made widely available; given whole genome data is involved, re-identification may be possible, as discussed in the ‘Anonymization’ subsection above. Multigenerational genomic datasets could even reveal cases of misattributed paternity, leading to substantial disruption and psychosocial harm to families.

There may also be broader social risks, depending on the type of research being conducted. As previously noted, attempts to use genetic markers for racialized therapeutic marketing approaches could contribute to the problematic reification of race. In addition, published research results could be stigmatizing to certain groups if they feed into existing ethnic or racial stereotypes. This happened most famously in the case of the Havasupai Tribe, where DNA gathered originally to study diabetes was used for other purposes, including delicate issues of mental health and migration, without properly attending to the values and sensitivities of members of the Tribe (de Vries et al. 2012).

### Identifying Relevant Values and Conflicts Among Them

#### Public Benefit, Harm Minimization and Justice

Since the proposal is to share genomic data with researchers outside the hospital, the potential public benefits are wide in scope. Analysis of the hospital’s genomic data (either on its own or in conjunction with other data sources) may be used to draw insights of relevance to much wider populations. While the aggregate benefit of such studies could be quite large, it is not clear whether the benefits to individual hospital patients who contributed data are substantial enough to outweigh the risks involved. There may be a trade-off, then, between preventing harm to hospital patients, and maximizing the public benefit generated from the use of their data. Some of these harms may be characterized as injustices, as explicated above, which may give rise to special reason to reduce or prevent them.

#### Engagement

Achieving a balance between those two values of harm minimization and promotion of public benefit would be one of the central concerns of a data sharing project like this. However, that balance crucially relies on an estimation of the interests of a diverse group of stakeholders. Hospital administrators may have some idea of what those interests are, but direct engagement with stakeholders would be valuable in clarifying their content and the relative weight individuals place on them. Engagement strategies of the sort described in Xafis et al. (2019) could be used here.

#### Transparency

While in this setting transparency about the data sharing plans might be achieved by obtaining prospective consent from hospital patients, this would be limited in nature. Since particular aims of future researchers are not identifiable at the point of clinical care, consent would have to be broad—discussing the general conditions of sharing, scope of potential research and what level of oversight and governance would be used. While such consent may have some moral force in authorizing the use of the data (Sheehan 2011), its limitations concerning the specificity of how patients’ data emphasize the need for the hospital to carefully adjudicate the risks and benefits of allowing the data to be shared (Hofmann 2009).

Transparency can also be achieved in other ways. Insofar as the interests of a variety of stakeholders are at stake, the hospital could explain how it came to a decision in this matter (for example, as an online resource). Ongoing uses of the data—with whom the hospital is partnering, for what research purposes, etc.—could be disclosed on the same platform, particularly so patients are made aware of the extent to which data about them is being shared and used.

#### Reflexivity

Solutions to these challenges would have to be reflexive, taking into account the impact of its decisions and seriously evaluating whether the data’s distribution actually end up generating the anticipated benefits. In the context of this case, reflexivity will be particularly relevant as the legal, social and cultural context around genomics changes. The risk of genetic discrimination, for instance, will be contingent on local laws and regulations that limit the use of the information in insurance and employment, while the risk of stigmatization may shift along with attitudes towards subgroups. At the same time, any purported benefits of research should not be taken for granted; reflexively would also involve evaluating the progress of research and adjusting sharing approaches as needed.

### Identifying Actions That Could Be Taken, Then Weighing Up the Relative Ethical Merit of the Options

#### Declining To Share Any Genomic Data with Researchers

The most straightforward option is for the hospital to enact a policy where no genomic data will be made available for secondary data research. The fewer people and institutions that have access to the data, the lower the risk of a breach. This option prioritizes a certain sort of harm minimization over public benefit: the hospital would refrain from exacerbating the risk of a data breach, and shut off the possibility of insensitive research. It also has the ancillary benefit of being more economical for the hospital, insofar as there would be no need to spend resources establishing rigorous mechanisms to ensure data is shared responsibly. But if we view harm minimization more broadly, this policy could be seen as problematic: the hospital is forgoing the opportunity to prevent harm to society more broadly, by making valuable genomic data available for analysis that could contribute to the development of beneficial treatment regimes.

#### Sharing Data with Certain Researchers

A policy of making the genomic data available for researchers would instead prioritize the potential public benefits of the genomic data. This is incredibly rich information that is going to be gathered anyway. This option is much more complex than the preceding, though, as it would require setting up a robust system to determine what would be shared (raw whole genome? Smaller chunks? Linked to clinical outcomes?), in what form (identifiable, anonymous, pseudonymous?), who would receive the data (specified local researchers? International data repositories?), what criteria would be put on use (only certain kinds of research? Only not-for-profit? Must have a collaboration with the hospital?) and what protections are in place (what data security protocols? What infrastructure to prevent re-identification? What level of ethics committee approval is needed?). Addressing each of these would be necessary to ensure that harms are adequately minimized and public benefits maximized.

#### Select the Option with the Most Ethical Weight and Communicate It to Stakeholders

For illustrative purposes, let us examine what an argument for the second option—sharing the data—could look like:

Sharing of clinical genomic data with researchers has an incredibly favourable risk-benefit ratio. There are risks of a breach, and anonymity of genomic data cannot be guaranteed, but these are manageable with appropriate security protocols and firm institutional rules against re-identification. Even if a breach occurred, it is unlikely individuals would face substantial genetic discrimination, as it is likely only experts in genetics would be in a position to interpret and draw meaningful conclusions from the breached data. And the cases of genetic discrimination that have been described to date originated from patients disclosing the information, not insurers or others learning about the information from a breach. And further risks of group harm can at least be mitigated by not sharing racial or ethnic details of patients. The benefits of sharing are admittedly somewhat nebulous—we should be wary of overhyping the promise of precision medicine. Nevertheless, genomic data does have real potential to improve certain areas of treatment and care, and even modest potential benefits can justify the relatively minimal risks of sharing.

This argument is not decisive on its own. It requires further empirical investigation, particularly surrounding the claim that risks of sharing are indeed minimal. It could be, on the contrary, that genomic data breaches can cause substantial harm as analytics of genomic data becomes more straightforward. And even if the risk-benefit ratio is favourable now, that could change over time—and so reflexivity demands that, if the hospital embarks on a policy of sharing, this policy be periodically revisited.

A decision against sharing data would still need to meet the standard of transparency. In light of the potential public benefit, the hospital should be open in its deliberation process and also be clear to researchers who query them as to their reasons. This could help prevent accusations that the hospital was acting primarily in self-interest, to keep potential innovations to themselves, when in fact a reasonable case can be made against sharing data based on the interests of patients themselves.

This option would also require further consideration of the numerous questions relating to governance and management just discussed. For example, one strategy to minimize the risk of re-identification would be for the hospital could host and provide data access in a secure environment (i.e. people cannot download the data and link it to external data sources unless they hack the system and download it). Such a system would be expensive, however, and so there would be a trade-off between cost containment and risk management. Full exploration of these further issues are beyond the scope of this paper, but we hope the framework presented in Xafis et al. (2019) and applied in this article would be of use in those deliberations.

## Conclusion

The realization of the promise of precision medicine requires the collation and analysis of substantial amounts of big data. Part of that process should involve critical reflection not just on what innovations can be generated but also how the benefits will be distributed fairly among the population. The same applies to the potential harms from the data use; anonymization is becoming more limited as a tool for mitigation, and in any case, there are potential ramifications for groups if results are poorly framed and reinforce objectionable stereotypes. The large role of genetics in precision medicine is also relevant, as data may reveal information about family members who are not technically part of a given dataset, and the spectre of genetic discrimination looms—especially in jurisdictions that currently lack, or have incomplete, anti-discrimination frameworks. Attending to shared values of the sort highlighted here can help stakeholders grapple with these issues and contribute towards earning public trust in the enterprise of precision medicine.
